# Identification and characterization of a novel Sso7d scaffold-based binder against Notch1

**DOI:** 10.1038/s41598-017-12246-1

**Published:** 2017-09-20

**Authors:** Tenzin Gocha, Balaji M. Rao, Ramanuj DasGupta

**Affiliations:** 1 New York University Langone Medical center, Perlmutter Cancer Center, Deparment of Biochemistry and Molecular Pharmacology, 522 1st Ave., Smilow Research Building, Rm 1206, New York, NY 10016 USA; 2 Department of Chemical and Biomolecular Engineering, North Carolina State University, Raleigh, North Carolina 27695 United States; 3 Genome Institute of Singapore, Cancer Therapeutics and Stratified Oncology, 60 Biopolis Street, #02-01, Genome, 138672 Singapore, Singapore

## Abstract

Notch signaling has important functions in regulating cell growth and development, misregulation of which has been implicated in various cancers. Monoclonal antibodies (mAbs) targeting Notch protein activity have already moved into clinical trials. However due to the limitations associated with cost and productivity of mAbs, there has been a surge in the development of complementary approaches that are based on non-antibody scaffolds. Non-antibody scaffolds are small proteins that are stable and can be engineered to develop high-affinity binders against specific targets of interest. Here we describe the isolation and characterization of a novel Notch1-binding protein, N9, obtained by screening of a combinatorial library based on the ultra-stable Sso7d scaffold. N9 targets the extracellular EGF-like repeats (ELR) 11–13 in Notch1, and therefore serves as a competitive inhibitor for Notch ligands to decrease expression of Notch target genes. We demonstrate that N9 recognizes surface expression of Notch1 on the plasma membrane and binds preferentially to cell lines misexpressing Notch1. Although N9 was selected against Notch1, we also observe cross-reactivity against other Notch receptors, including Notch2/3. Finally, we demonstrate that N9 inhibits proliferation and generation of tumorspheres in Notch expressing cancer cell lines, suggesting its potential as a therapeutic agent in Notch-associated malignancies.

## Introduction

Cell signaling constitutes a multitude of highly regulated protein-protein interactions (PPIs) through which cells communicate with each other. Misregulated cell signaling leads to disease, including cancer^1^. It is therefore important to develop novel molecular tools to disrupt specific PPIs that can help elucidate the nature and function of individual PPIs in modulating cell signaling, and subsequently cellular phenotypes. Furthermore, such tools have the potential to translate into efficient therapeutics. However, the ability to specifically inhibit a PPI in a given context remains challenging. Commonly used tools such as genetic knockouts or siRNA-mediated knockdown of protein expression cannot selectively target specific PPIs^2^. Additionally, the use of genetic manipulation to introduce targeted mutations or truncations may affect overall protein stability and confound studies on PPIs. Screening of small molecule libraries have resulted in identification of PPI-inhibitors. However, the use of these compounds is limited by their poor target specificity and toxicity^3^. Biologics (protein based drugs) on the other hand, exemplified by monoclonal antibodies (mAbs), have been very useful in perturbing PPIs and its success has been reflected in its recent adoption by the pharmaceutical companies in their drug portfolio^3–6^. Although mAbs have been very successful, antibodies have certain limitations based on their size (~150 kD), stability, and cost of production that restrict their large-scale adoption for various applications^7–10^. Due to these limitations, more recently alternative strategies have been adopted to develop affinity reagents^11^. This has led to a series of transitions from mAbs to Fabs (Fragment antigen binding) and then to scFvs (single chain fragment variable)^6,10,12^. ScFvs are approximately 7 times smaller than mAbs and have been shown to have added advantages of stability and productivity^10^.

**Figure 1:**
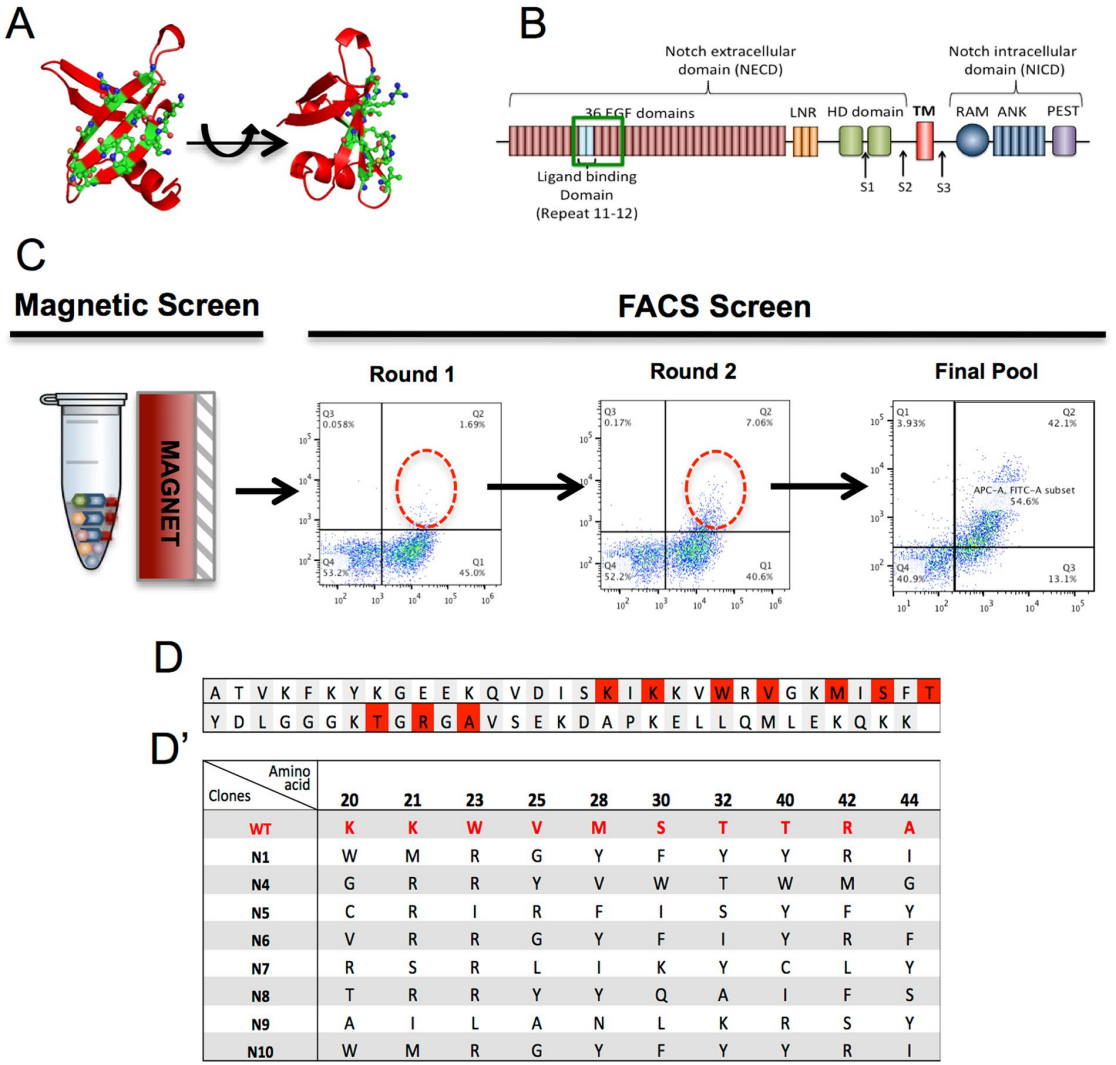
Screening for Sso7d variants against Notch1 ELR 11–15. (**A**) Crystal structure of Sso7d (PDB ID: 1SSO^43^) with randomized residues show in green sticks. Note: Image generated using Pymol. (**B**) Domain organization of Notch1. Green box highlights the ELR 11–15 domain of Notch1 chosen to screen for Sso7d-binders. (**C**) Schematics of Yeast Surface Display screen. After one round of magnetic screen with biotinylated-protein-bound magnetic beads (Dynabeads Biotin-binder), two rounds of selections were performed using Flow cytometry. Yeast isolated from magnetic screen, and two rounds of FAC sorting were double stained with 0.2 μM ELR 11–15 and anti-Myc antibody. Red circles depict enriched populations (**D**) Amino acid sequence of sso7d with randomized residues marked in red. (**D’**) Amino acid sequences of the candidate hits. Only the randomized residues are shown.

More recently, protein engineering technologies have enabled the use of smaller and synthesizable non-antibody scaffolds for generating binders with high affinity and specificity^4,10,13–15^. Because of small size, robust protein scaffold, and the ease of recombinant expression in bacterial platforms, there is a wide-spread interest in binders based on non-antibody scaffolds for research as well as theranostic applications^7,14^. In this study we describe the identification of Notch1 specific binders by screening a combinatorial library obtained by mutagenesis of the Sso7d protein scaffold using yeast surface display (Fig. 1A)^16^. Sso7d is an ultra-stable 7 KDa protein from the hyperthermophilic archaeon *Sulfolobus solfactaricus* and has been shown to be a versatile scaffold for the generation of binders to a diverse range of target proteins^9,16–18^. Notch is a receptor mediated signalling pathway, the dysregulation of which has been implicated in various diseases including cancer^19^. The binding of ligands, Jagged1/2 (JAG1/2) and Delta-like 4 (DLL4), to the Notch receptor is known to trigger a series of proteolytic cleavages that eventually results in the generation of the Notch Intracellular domain (NICD). NICD translocates to the nucleus, where it binds to Mastermind and CSL (CBF1, Suppressor of Hairless, Lag-1) to activate transcription of target genes^20–22^. The extracellular domain of Notch comprises of EGF Like Repeats (ELR) that have been shown to be critical for ligand binding^23,24^ (Fig. 1B). Here we report the identification and functional characterization of an Sso7d variant, called Notch binder clone-9 or N9. We demonstrate that N9 binds to cell-surface expressed Notch1 and that binding of N9 to Notch1 inhibits its interactions with cognate ligand, such as JAG1 and DLL4. Consequently, N9 inhibits proliferation of Notch1-expressing breast cancer cell lines and downregulates expression of Notch target genes. Finally, N9 effectively reduces tumorsphere forming ability of breast and colorectal cancer stem-like cells, a property that has been shown to rely on active Notch signaling^25,26^. Our results highlight the potential use of Sso7d as a non-antibody scaffold for the modulation of cell signaling by perturbing specific ligand-receptor interactions.

## Results

### Screen for sso7d binders to Notch1 ELR 11–15

Binders specific to Notch1 were isolated from a yeast display library of Sso7d mutants (~10^8^ diversity), which was previously evaluated for its ability to generate high affinity ligands to different protein targets^13,16^. To identify binders that can interact with Notch1 and possibly modulate its ligand binding activity, we screened the Sso7d library against purified Notch1 ELR 11–15 region that constitutes the ligand binding domain^25,27^. Magnetic sorting and two rounds of FACS was used to isolate a pool of binders with the highest affinity for Notch1 **(**Supplementary Fig. S1, Fig. 1C **)**. 10 clones were randomly picked from this pool; DNA sequencing identified 8 unique clones **(**Fig. 1D **)**. Of these, we picked four individual clones for further characterization.

### N9 binds to Notch1 on the cell surface

**Figure 2:**
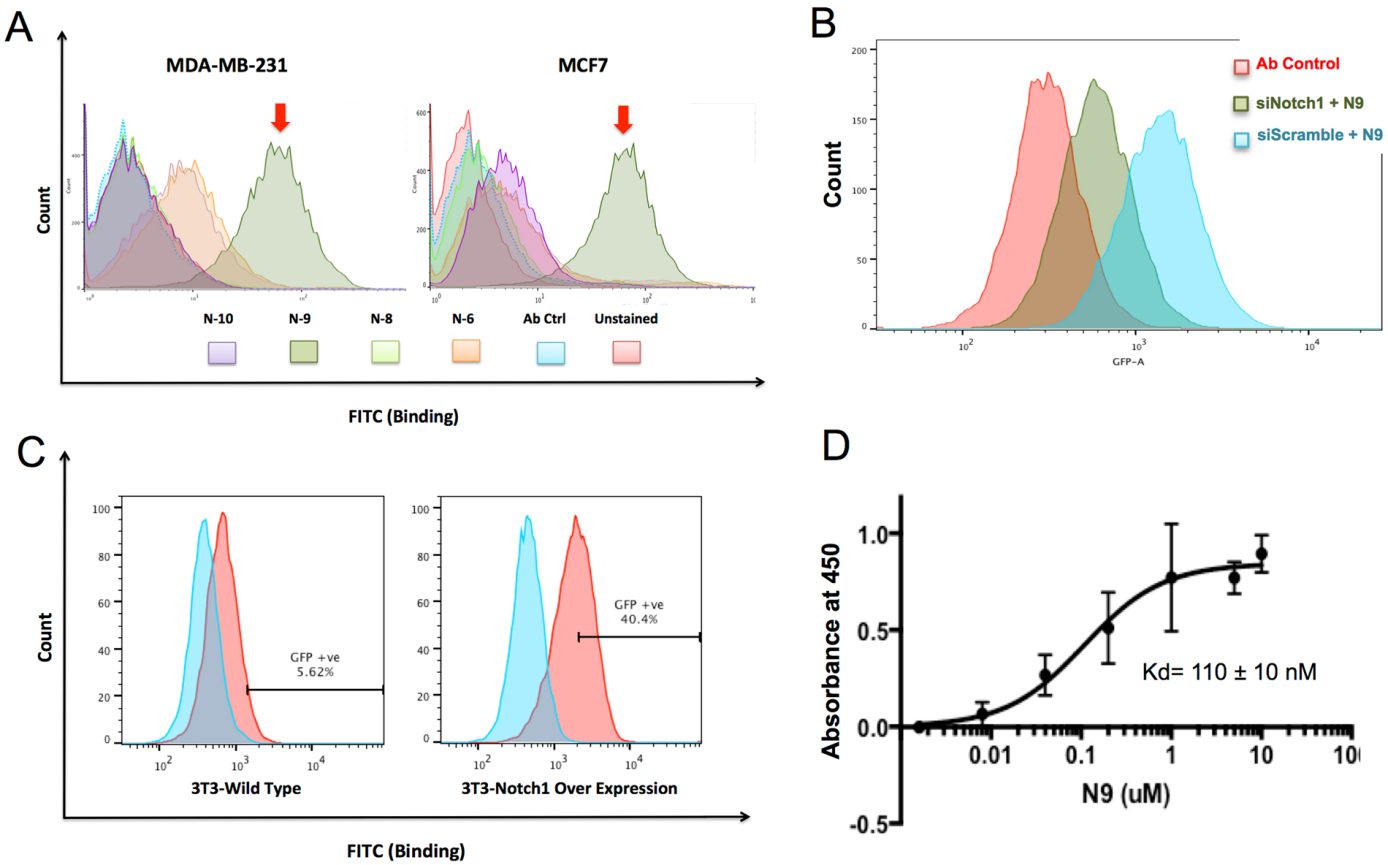
Characterization of Sso7d Notch1 binders. (**A**) Cell surface binding of candidate hits. MCF7 and MDA-MB-231 cells were stained with 1 μM candidate hits and labeled with anti-Myc-488 antibody. Red arrows indicate N9. (**B**) Notch1 knockdown decreases N9 binding. MCF7 cells, either transfected with siNotch1 or scramble control were stained with 1 uM N9 followed by Anti-Myc-488 antibody. Notice the shift of fluorescence peak intensity from scrambled control (blue) to siNotch 1 (green). (**C**) Notch1 over expression increases N9 binding. 3T3 wild type or 3T3-hNotch1 cells were incubated with 50 nM N9 and labeled with anti-Myc-488 antibody. Note the increase in N9 binding from ~5.6% in 3T3 wild type to 40.4% in 3T3-hNotch1 cells. (**D**) Direct binding assay of N9 and Notch1 ELR 11–15. ELR 11–15 coated wells were incubated with indicated concentrations of N9 and probed with anti-Myc-HRP antibody. Kd estimate of 0.1 ± 0.01 μM.

Four individual binding clones were recombinantly expressed in E.coli and purified proteins were used to assess binding to NOTCH1 expressing cell lines. Because Notch1 has been shown to be overexpressed in breast cancer^28^, we used two well-studied breast cancer cell lines, MCF7 and MDA-MB-231 for the binding assay. Even though all candidate binders displayed some degree of binding to the two cell lines, clone Notch1-binder 9 (N9) exhibited the most robust binding **(**Fig. 2A **)**, and was therefore chosen for further analysis.

In order to confirm the specificity of N9 binding to endogenous Notch1, we performed siRNA-mediated knockdown of Notch1 in MCF7 cells. Knockdown of Notch1 significantly reduced binding of N9 to MCF7 cells (Fig. 2B, Supplementary Fig. S2A **)**. Conversely, in gain-of-function studies, we observed increased N9 binding to 3T3 cells overexpressing Notch1 (3T3-hN1) compared to the control parental wild type line (3T3-WT) **(**Fig. 2C **)**. We subsequently measured the binding affinity of N9 using the 3T3-hN1 cell line. N9 bound to 3T3-hN1 with an affinity (Kd) of 300 nM **(**Supplementary Fig. S2B-C **)**. Notably, in ELISA-based binding assays *in vitro*, N9 displayed binding to purified ELR 11–15 with affinity of 110 ± 10 nM **(**Fig. 2D **)**. Strikingly, although the yeast display screen was performed against Notch1 ELR 11–15, we observed cross-reactivity of N9 to other Notch receptors (Notch2/3) as well **(**Supplementary Fig. S3 **)**. Taken together, these results suggest that N9 can interact with Notch1 and that Notch1 expression is at least partly necessary as well as sufficient to confer N9 binding (Fig. 2B,C; Supplemetary Fig. S2A).

### N9 binds to ELR 11–13 of Notch1 and competes with ligand binding

**Figure 3:**
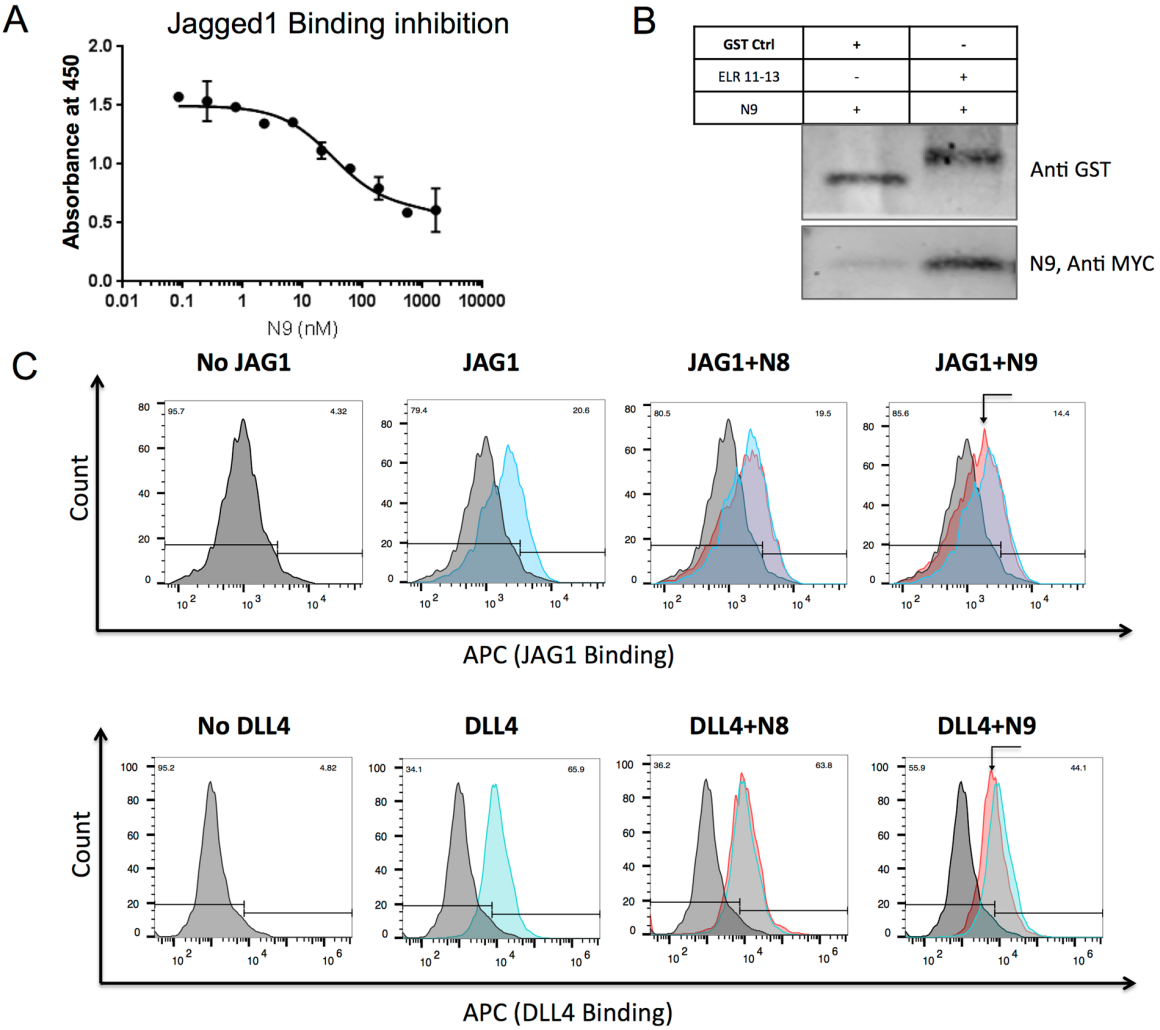
N9 binds to ELR 11–13 and inhibits Notch ligand binding. (**A**) N9 inhibits Notch1/Jagged1 binding. ELR 11–15 coated wells were incubated with indicated concentrations of N9 and 100 nM Jagged1-Fc. Binding was measured using Protein-G HRP conjugate. (**B**) N9 binds to ELR 11–13 of Notch1. ELR 11–13 or a GST tagged control protein were incubated with N9 and pulled down using GST beads. (**C**) N9 inhibits Notch ligand binding. HEK 293 T cells transiently transfected with hNotch1 were preincubated with binders and 2 nM JAG1-Fc and DLL4 were added. Binding was detected using Anti-Fc APC antibody. Note that N9, but not N8 (scaffold control), decreases Notch ligand binding.

Since N9 was screened against ELR 11–15, which constitutes the ligand binding domain of Notch1, we first tested the ability of N9 to compete for binding with one of its ligand, JAG1. In competitive ELISA, N9 inhibited Jagged1 binding to ELR 11–15 in a dose-dependent manner, Ki ~70 ± 20nM **(**Fig. 3A **)**. It has previously been shown that ELR 11–13 is sufficient for interaction between Notch1 and its ligands, and that it constitutes the core recognition site^23–25,29^. Therefore, to map the binding domain of N9 on Notch1 we generated a shorter construct constituting ELR 11–13 and performed GST pull down assays. As expected, ELR 11–13, but not the GST control, pulled down N9 efficiently **(**Fig. 3B **)**. Finally we tested whether N9 can inhibit Notch ligands JAG1 and DLL4 binding on cell surface. As a scaffold control, we evaluated N8, which didn’t bind to the breast cancer cell lines as strongly as N9 **(**Fig. 2A **)**. N9, but not N8, reduced binding of both JAG1 and DLL4 to HEK293-hN1 **(**Fig. 3C and Supplementary Fig. S4 **)**. Thus, our results confirm that N9 binds to EGF like repeat 11–13 of Notch1 and competes with binding of Notch1 to its cognate ligands.

### Functional characterization of N9 in cell based assays

**Figure 4:**
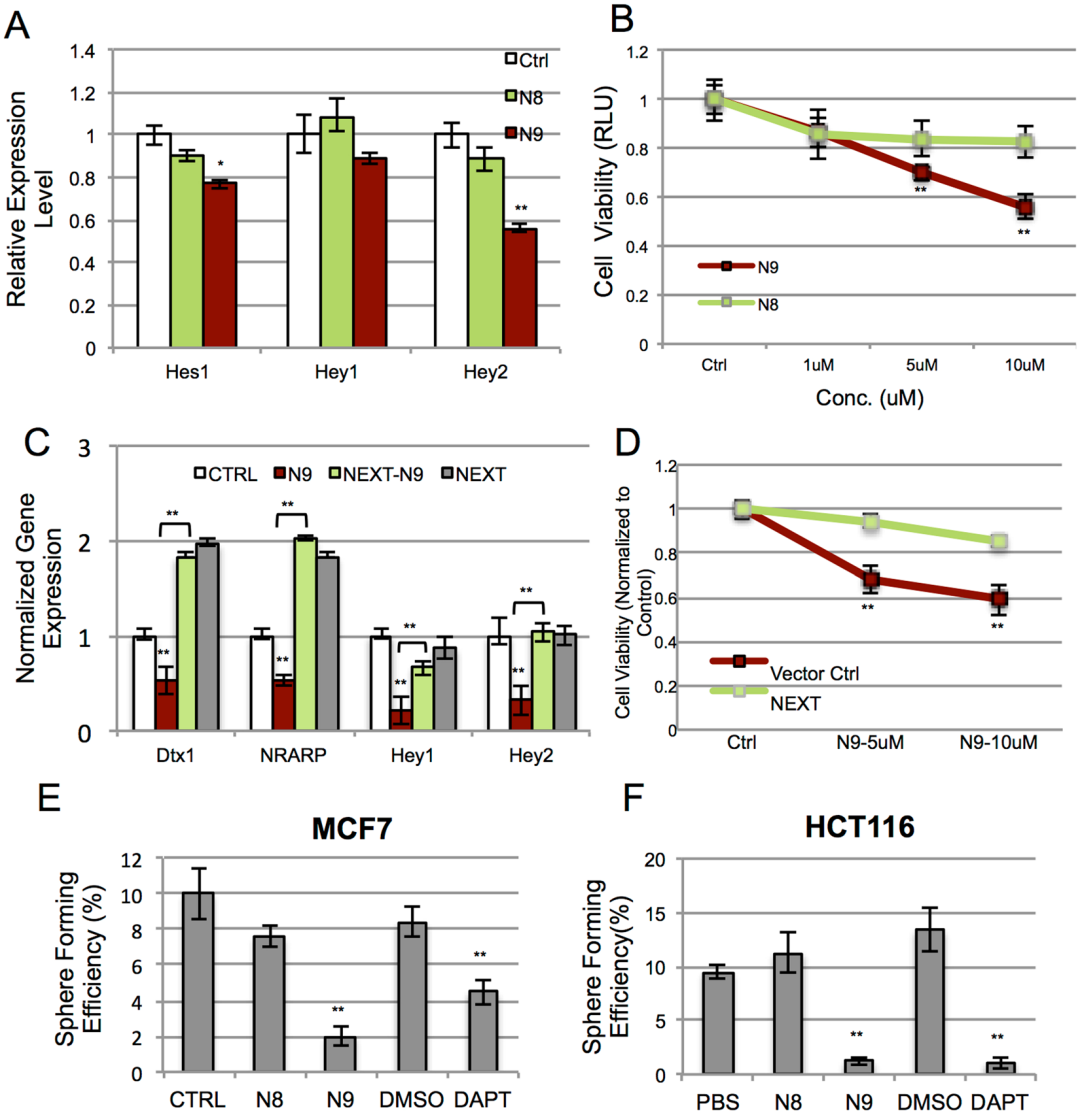
N9 decreases Notch pathway activity and abrogates growth of tumorspheres. (**A**) N9 (red bars) decreases expression of Notch target genes compared to scaffold-control N8 (green bars), and buffer control (white bars). (**B**) N9 decreases MCF7 cell viability (red line), compared to N8 scaffold control (green line). MCF7 cells were incubated with indicated concentration of binders for three days and cell viability was measured. (**C**,**D**) N9-mediated decrease in expression of Notch target genes **(C)** (red bars) and cell viability (**D**) (red line) can be rescued by misexpression of extracellularly truncated Notch (NEXT) (green bars in **C**, and green line in **D**). (**E**,**F**) N9 decreases tumorsphere formation. Single cell suspensions of MCF7 and HCT116 cells were cultured with 5 μM binders or 10 μM DAPT for 5 days and spheres bigger than 20 um diameter were counted. Note that N9, but not N8 (scaffold control), decreases tumorsphere formation. (*P < 0.05, **P < 0.01).

**Figure 5:**
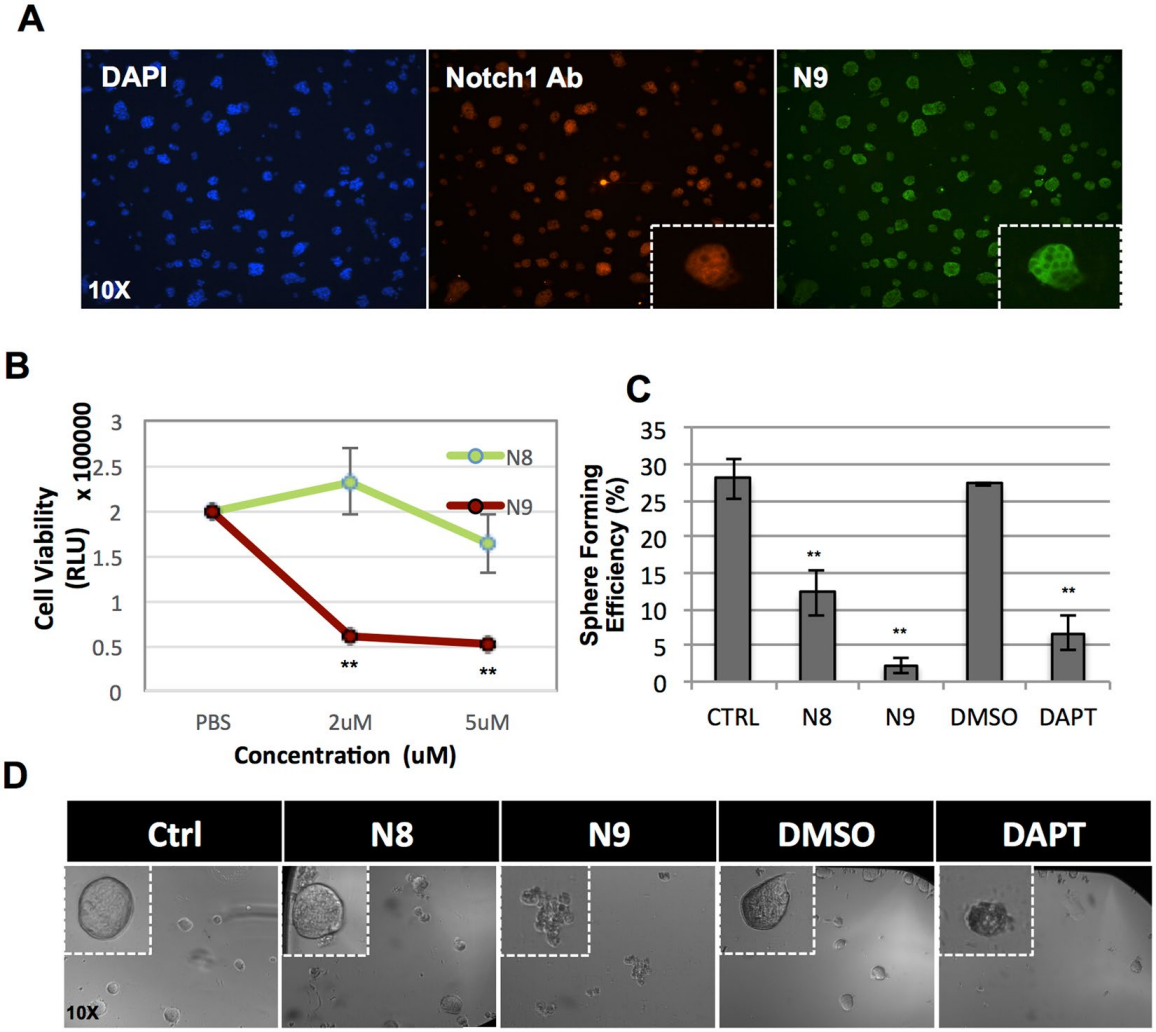
N9 decreases tumorsphere growth of patient derived cell line. (**A**) Patient derived colorectal cancer cell lines (PDCRCs) stained with commercial Notch1 antibody (red), N9 (green), and the nuclear stain DAPI (blue). Inset depicts magnified view of a representative colony. Note the similarity in staining pattern between commercial antibody and N9. (**B)** N9 (red line) decreases PDCRC cell viability. PDCRC cells were incubated with indicated concentration of binders for three days and cell viability was measured. **(C)** N9 decreases PDCRC tumorsphere formation. Single cell suspension of PDCRC cells were treated with 5 μM binders and 10 μM DAPT for five days and spheres bigger than 20μm were counted. **(D)** Representative images of PDCRC spheres. Inset depicts magnified view of a representative sphere. (*P < 0.05, **P < 0.01).

As N9 bound to ELR 11–13 and competed with ligand binding, we investigated whether it can modulate Notch1 pathway activity *in vitro*. Consistent with effective inhibition of JAG1 and DLL4 binding, addition of purified N9 to MCF7 cells significantly decreased expression of Notch target genes, including Hes1, Hey1 and HeyL **(**Fig. 4A **)**. Since the Notch pathway has been reported to inhibit growth of breast cancer cell lines, we assessed whether N9 could affect proliferation of breast cancer cells. MCF7 cells were incubated with increasing concentrations of binders, and cell viability measured after 72 hours. Notably, N9 inhibited proliferation of MCF7 cells in a dose-dependent manner **(**Fig. 4B **)**. Additionally, N9 treatment did not significantly affect the proliferation of control non-cancerous HEK293 cells **(**Supplementary Fig. S5 **)**. Importantly, N9-mediated inhibition of Notch target gene expression and reduced cell viability could be rescued by misexpression of an extracellular-domain truncated Notch (NEXT). These data suggest that the N9 phenotype is specifically dependent on its ability to modulate Notch signaling activity **(**Fig. 4C-D and Supplementary Fig. S6A-B **)**. Notch pathway is implicated in cancer stem cell (CSC) maintenance and inhibition of Notch1 signaling has been reported to decrease growth of tumorspheres^25,26^. Therefore we investigated whether N9 could regulate the self-renewing capability of CSC-like cells. We cultured colorectal cancer (HCT116), breast cancer (MCF7) and patient-derived colorectal cancer cell lines in serum-free tumorsphere media in the presence or absence of binders. As shown in Figs 4E–F and 5, N9 significantly reduced tumorsphere formation to an extent similar to that of the known Notch1 inhibitor, DAPT. Altogether, these observations suggest that N9 can robustly target self-renewing ability of CSC-like cells, likely by blocking activity of the Notch pathway.

**Figure 6:**
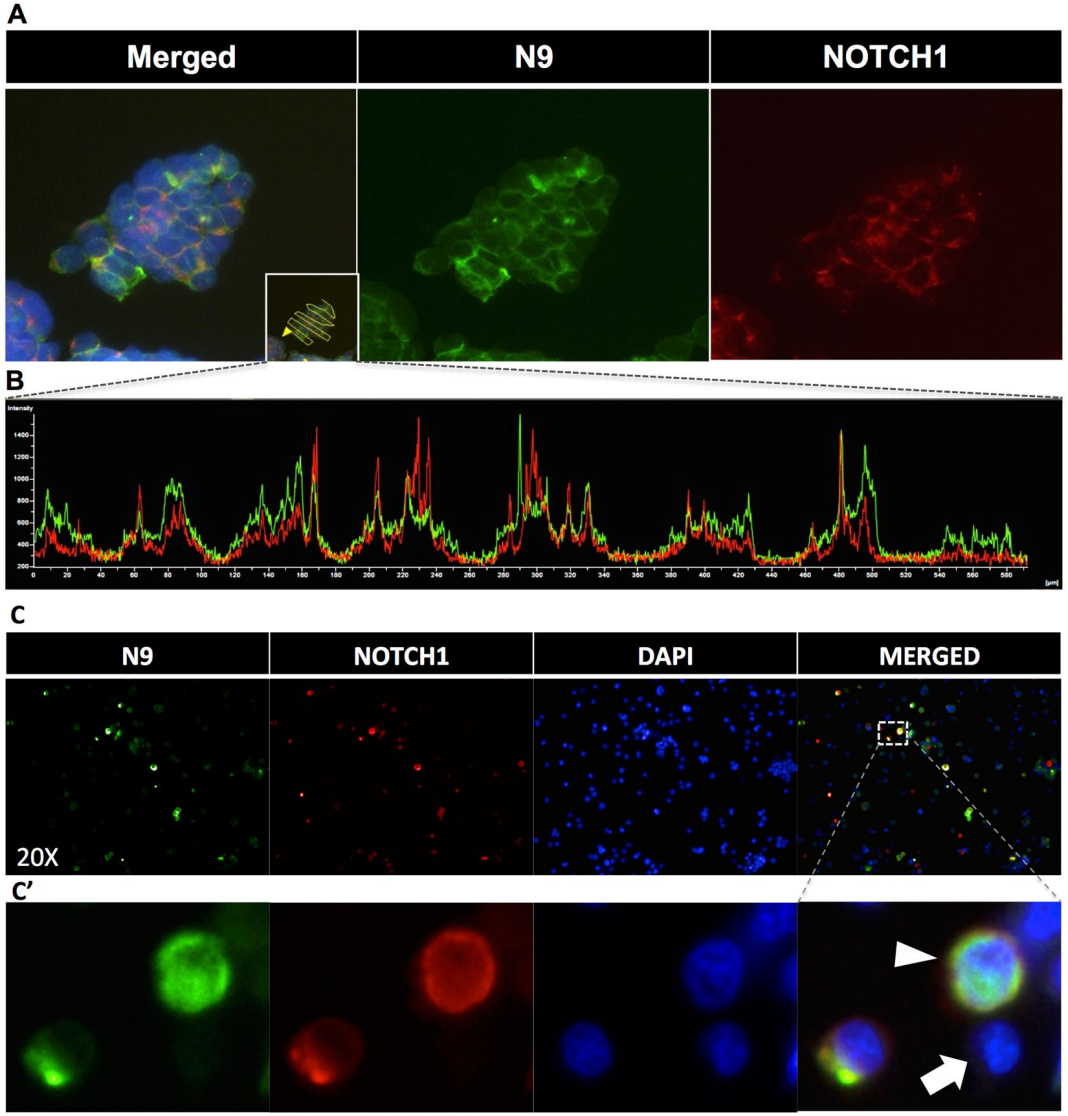
Evaluation of N9 in immunostaining application. (**A**) HCT116 cells were stained with N9 (green), commercial Notch1 antibody (red) and DAPI (blue). Note the staining at cell-cell interfaces. (**B**) Fluorescence intensity profile of N9 and Notch1 staining of (**A**). Note the similarity in pattern of the green (N9) and red (Notch1) curves indicating co-localization of the signals. (**C**) HEK293 cells were transiently transfected with Notch1 and stained with N9 (green), commercial Notch1 antibody (red) and DAPI (blue). (**C’**) Magnified view of the inset in (**C**) showing staining of Notch1-transfected (arrowhead) and non-transfected (arrow) cells. Note that only Notch1 transfected cells (arrowhead) were stained with N9.

We also evaluated whether N9 can be employed for immunofluorescent applications. Corroborating our previous data, N9 but not N8 effectively labeled MCF7 cells **(**Supplementary Fig. S7 **)**. Furthermore, when compared to a commercially available Notch1 antibody, we observed significant co-localization at the cell membrane with very little staining at free surfaces. (Fig. 6A). Finally, we transiently transfected HE293 cells with Notch1 and stained with either N9 or the commercially Notch1 antibody. As expected, strong colocalization was observed in cells expressing Notch1. Notably, no staining was observed in untransfected cells **(**Fig. 6C-C’ **)**.

## Discussion

The last few decades have witnessed a surge in FDA-approval for protein-based drugs, or biologics^5^. Despite the huge success of monoclonal antibodies, limitations based on size, post-translational modification, stability, productivity, and cost-effectiveness have restricted their applicability^10,11,30^. Considering these constraints, the use of small, non-antibody scaffolds, such as Sso7d have the potential to serve as complementary and robust alternative solutions in the domain of next-generation biologics.

In this study we demonstrate the identification and functional characterization of an Sso7d variant, called N9 that binds to Notch1 with an equilibrium dissociation constant in the ~100 nM range. The dissociation constant for N9 obtained from direct ELISA and and JAG1 competition assay were similar, suggesting specific binding of N9 to the Notch1 receptor. Although binding site of JAG1 on Notch1 receptor is well studied and has been narrowed down to ELR 11–12 of Notch1, additional regions (ELR 6–15) have also been implicated in binding^31,32^. The fact that N9 bound to ELR 11–13 and competed with JAG1 suggests that ELR 11–13 is sufficient for binding and hence corroborates previous reports that ELR 11–12 contain the core recognition element of Notch/JAG1 interaction^24,29^.

Despite binding of N9 to Notch1 on the of MCF7 cell lines, we noticed that knockdown of Notch2 also decreased N9 binding on cell surface.**(**Supplementary Fig. S8 **)**. Upon further investigation, we observed that knockdown of Notch receptors 1–3 generally decreased staining for N9 **(**Supplementary Fig. S3 **)**. Since ELR 11–13 of Notch1–3 exhibits ~70% sequence similarity, our data suggests that N9 binds to an epitope that is common to all these proteins. Previous studies have shown that Notch receptors can all bind JAG1, which suggests presence of common epitopes among Notch receptors^33,34^. Moreover, it has also been reported that monoclonal antibodies generated against one specific Notch receptor show cross-reactivity against other Notch receptors^35,36^. Knockdown of Notch4 did not reduce N9 staining **(**Supplementary Fig. S9 **)**. It is noteworthy that ELR 11–13 of Notch1 only has 50% sequence similarity with that of Notch4. If needed, paralog specificity for candidate binders can be attained by conducting negative selection screens against each paralog, prior to positive selection against the paralog of interest.

Notably, the ability of N9 to downregulate Notch pathway activity, decrease proliferation of Notch-dependent cancer cells, and block the formation of tumorspheres highlights the promise of these binders to be developed as putative cancer therapeutic agents. It should be noted that these Notch1 binders were isolated from a naïve Sso7d library, without any further affinity maturation. Therefore it is conceivable that additional rounds of mutagenesis and screening may result in isolation of binders with significantly higher affinity. Our findings also confirm previous reports that blocking Notch1 function inhibits self-renewal capacity of CSC-like cells^25,26^. The importance of the findings in this study is underscored by the fact that antibodies against Notch pathway are being tested against different cancers in clinical trials as CSC-targeted therapy^37^. Therefore the discovery and characterization of small biologics-based Notch inhbitors could add to the rapidly evolving pipeline for Notch-targeted drugs.

One of the additional potential application of small protein binders is in the field of imaging and diagnostics as illustrated by the success of radio-labelled affibodies^38^. The small size aids in enhanced biodistribution and faster clearance leading to increased signal-to-noise ratio^39^. In this report, we demonstrate that N9 can effectively label cells for immunostaining and flow-cytometry applications. These observations support previous reports that Notch1 mostly accumulates at cell-cell interface rather than free surfaces^40^. Future studies will be aimed at developing paralogue-specific Notch binders and optimizing them for therapeutic use and/or as molecular probes to study the function of Notch signaling in tumor progression and CSCs.

Finally, this study exemplifies the qualities of Sso7d as a scaffold protein to develop binders against specific target proteins. We provide proof-of-concept for the use of Sso7d as a non-antibody scaffold for development of reagents that can modulate and/or monitor oncogenic signaling pathways. Sso7d as a fusion protein or binder has already been used for development of a variety of molecular tools, including the high fidelity DNA polymerase^41^ and ELISA reagents. The fact that it could be used to perturb cell signaling networks by modulating PPIs opens a whole new era of application and opportunities.

## Material and Methods

### Cell lines and medium

MDA-MB-231 and HCT116 cell lines were bought from ATCC and MCF7 cell lines was a gift from Michael Garabedian’s laboratory (New York University School of medicine, New York, NY). 3T3 and 3T3-hN1 cell lines were gifted by Ianis Aifantis’s Laboratory (New York University School of medicine, New York, NY). HCT116 was cultured in McCoy’s 5 A medium with 10% FBS and P/S. MCF7 and MDA-MB-231 was cultured in DMEM with 10% FBS and P/S.

### Plasmids, Cloning, protein expression and purification

Notch1 ELR 11–15 construct was a gift from Dighe Lab, Indian institute of Science. The plasmid was transformed into BL21 cells and purified using GST beads and s200 size exclusion chromatography. ELR 11–13 was made by mutating pGEX-4E ELR 11–15 construct by site-directed mutagenesis using Quikchange Site directed mutagenesis kit (Agilent) and confirmed by Sanger sequencing. hNotch1 construct was kindly provided by Professor Alison Banhan. NEXT construct was kindly provided by the Kopan Lab.

### Yeast display library Screening

The yeast display library of Sso7d mutants used for this study has been previously described^16^. The yeast display library was screened for binders to Notch1 using a combination of magnet-activated cell sorting and flow cytometry, as described^42^. Briefly, biotinylated Notch1 was incubated with 100μl of biotin-binder beads (4 × 10^8^ beads per ml, Invitrogen) for 2 hours. The library was subjected to a negative selection step by incubation with with naked beads, and then with beads coated with GST control protein, for 2 hours each at 4 °C; yeast cells binding to the beads were discarded. Subsequently, the unbound yeast were incubated with magnetic beads coated with Notch1 for 1 hour at 4 °C, and bead-bound yeast cells were expanded. This pool of cells after the magnetic selection steps was further screened using two rounds of fluorescence activated cell sorting (FACS) to isolate high affinity binders.. The first round of FACS was performed by incubating the library with 200 nM of biotinylated Notch1 for 1 hr at room temperature. For the second round of FACS, the pool of sorted cells from round 1 was labeled with 25 nM of biotinylated Notch1 and top population from 25 nM sort were collected. Collected populations were labelled with 200 nM of biotinylated Notch1 to validate binding.

Following the last sort, the pool of yeast cells was plated on an SDCAA plate. Plasmids from individual colonies were recovered using Yeast Zymoprep kit and transformed into DH5alpha E. coli cells. Isolated plasmids from DH5alpha were then sequenced.

### Recombinant Sso7d protein production

Sso7d variants from the screen were cloned into pET28a and transformed in BL21 cells for protein expression. BL21 cells were grown in 1 L LB till OD reaches 0.6–0.8 and induced with 1 mM IPTG at 25 degree overnight in a shaker incubator. Cells were lysed in 50 mM Tris, 250 mM NaCL, 0.5 mM TCEP with EDTA free protease inhibitor and centrifuged at 14,000 rpm for 20 minutes. Supernatant was incubated with 2 ml Ni-NTA agarose beads (Pierce) for 1 hour and eluted with 250 mM Imidazole in lysis buffer. Sso7d containing fractions were then pooled and purified further using Size exclusion chromatograpy s75 column with 10 mM HEPES, 150 mM NaCL. For cell culture work, protein was then dialyzed in PBS buffer using Slide-a-lyzer cassette (Pierce).

### Flow cytometry binding and competition assay

MCF7, MDA-MB-231 cells were washed with PBS and harvested using 1 mM EDTA in PBS. Detached cells were passed through 25 G syringe to ensure single cell suspension. Cells were incubated with binders for 1 hour at room temperature and stained with anti Myc-488 (Millipore clone 4A6) antibody for 1 hour on ice. For JAG1 and DLL4 competition assay, Notch1 transfected HEK293 cells were preincubated with 20 μM of binders for 1 hour on ice. 2 nM of human recombinant JAG1-Fc or DLL4-Fc (R&D Systesms) was added and incubated for another 1 hour. Cells were stained with APC conjugated Mouse Anti-Fc antibody (H2, ab99768) for half hour on ice and analysed using Amnis ImageStream Imaging flow cytometer.

### siRNA mediated knockdown

MCF7 cells were plated in 6 well plate and after 12 hours, transfected with 5 nM siRNA (Ambion silencer select) using RNAimax (Thermofisher) in complete media without Penicillin and Streptomycin. 48 hours after transfection, cells were harvested for flow cytometry or immunostaining.

### ELISA

50 ng of target protein was coated on Maxisorb plate overnight in carbonate buffer. After washing three times with PBS, plate was blocked with Pierce blocking buffer (Lot number) for 1 hour. Different concentrations of sso7d variants were added and incubated for 1 hour at room temperature. Plate was washed 3 times with PBST (PBS,0.05% Tween 20) and was incubated with 1:1000 dilution of Anti-myc antibody (Millipore clone 4A6) for one hour. After washing 5 times with PBST, wells were incubated with 1:5000 dilution of Goat anti-mouse HRP conjugate (Bethyl laboratories, A90–109P) or 1:50,000 dilution of protein G-HRP conjugate (Abcam, ab7460) for 1 hour. Wells were washed 5 times with PBST and 1-Step Ultra TMB (Thermofisher) ELISA substrate was added. After color development, the reaction was stopped using 2 M sulfuric acid and read at 450 using SpectraMax. Kd was determined using nonlinear regression, specific binding analysis in Graphpad Prism7. For JAG1 competition ELISA, wells were preincubated with serial dilution of N9 for 1 hour and 100 nM of human recombinant JAG1 Fc (R&D Systems) was added in each well. After one hour incubation, wells were incubated 1:50,000 dilution of protein G-HRP conjugate (Abcam, ab7460) for 1 hour. Wells were washed 5 times with PBST and 1-Step Ultra TMB (Thermofisher) ELISA substrate was added. After color development, the reaction was stopped using 2 M sulfuric acid and read at 450 using SpectraMax. Ki was determined using nonlinear regression, specific binding analysis in Graphpad Prism7.

### GST pull down

Equal amount of GST tagged proteins (~7 μg) were incubated with 20 μl GST beads for 1 hour at 4 degree. Bead-protein complex was incubated with the binders for another 1 hour at 4 degree and spun down. After several washes, protein was eluted either with 10 mM GSH or boiled with sample buffer (Biorad) and loaded on precast 4–15% gel (Biorad). Gel was transferred onto nictrocellulose membrane using Trans-Blot turbo transfer system (Biorad) and stained with Anti-GST (sc-138, Clone B14) or anti-Myc antibody (Millipore clone 4A6). Blots were detected with anti-mouse IR 800 secondary antibody using Odyssey imaging system (LI-COR).

### Collection and processing of patient derived colorectal cancer (PDCRC) cell line

Primary colorectal cancer tissue from a patient undergoing surgical resection was obtained from National Cancer Center, Singapore, with patient’s informed consent, as per the ethical guidelines and with approval from the Singhealth Centralized Institutional Review Board (CIRB). The tissue was aseptically minced and dissociated enzymatically using Collagenase and hyaluronidase at 37 °C into a cell suspension, which is then seeded onto plates pre-coated with Coating Matrix (Gibco). The cancer cells obtained are subsequently cultured in DMEM-F12 media supplemented with hEGF (20ng/mL), bFGF (10ng/mL) and 1% B27 without Vitamin A. Detailed protocol is available upon request.

### Immunostaining

HCT116, HEK293 or PDCRC cells were fixed with 4% Formaldehyde and permeablized with PBS + 0.5% Triton-X. Staining were performed over night at 4 degree in Odyssey blocking buffer (Licor). Antibodies used were Anti-Notch1, Cell signaling, D6F11 (1:200), Anti Myc-488, Millipore cl4A6 (1:1000), DAPI (1:1000) and imaged on Nikon TE2000-E microscope or PerkinElmer Operetta High-Content Imaging system.

### Cell proliferation and viability assay

MCF7, MDA-MB-231, HCT116, PDCRC or HEK293 cells were plated in 96 well plates at 5000 cells/well and binders were added after 12 hours at indicated concentrations. Cell viability was determined after 72 hours using Cell Titre Glo (Promega).

### qRT PCR

MCF7 or HCT116 cells were treated with 10 μM of binders for 72 hours. Cells were harvested and RNAs isolated from cell pellet using TRIzol reagent and purified using RNA cleanup kit (Qiagen). cDNA was prepared using High Capacity reverse transcription kit (Applied Biosystems) as per the manufacturer’s protocol. Real time PCR were carried on using Brilliant II SYBR green masters mix (Applied Biosystems) using MxPro-Mx3005 P system (Stratagene). Gene expressions were normalized to GAPDH.

### Notch rescue experiment

For NEXT (Extracellular-domain truncated Notch) mediated rescue experiment, HCT116 cells were grown in 6 well plate and transfected with 4ug of NEXT or vector control using lipofectamine 2000 (Thermofisher). Binders (10 uM) were added after 24 hrs and incubated for 72 hours. Gene expression and cell viability were measured as above.

### Tumorsphere assay

MCF7, HCT116 and PDCRC cells were washed once with PBS and harvested using 1 mM EDTA in PBS. Detached cells were passed through 25 G syringe three times to ensure single cell suspension. 1000 cells/well were plated in ultra-low attachment 96 well plates in serum free DMEM F12 media supplemented with B27, rEGF and rFGF and 0.3% Low melting agarose. 10 uM binders were added during cell seeding and spheres were imaged and counted after one week using Nikon TE000-E microscope, JOBS module (NIS Elements, Nikon).

### Statistical Analysis

Significance was determined using Student’s two-tailed T test based on at least three independent experiments, unless otherwise noted.

## Electronic supplementary material

Supplementary files

## References

[1] Martin, G. S. Cell signaling and cancer. *Cancer Cell* (2003). 10.1016/s1535-6108(03)00216-2 14522250

[2] Dreze M (2009). ‘Edgetic’ perturbation of a C. elegans BCL2 ortholog. Nat. Methods.

[3] Scott DE, Bayly AR, Abell C, Skidmore J (2016). Small molecules, big targets: drug discovery faces the protein–protein interaction challenge. Nat Rev Drug Discov.

[4] Feldwisch J (2010). Design of an optimized scaffold for affibody molecules. J. Mol. Biol..

[5] Weiner LM, Surana R, Wang S (2010). Monoclonal antibodies: versatile platforms for cancer immunotherapy. Nat. Rev. Immunol..

[6] Stumpp, M. T., Binz, H. K. & Amstutz, P. DARPins: a new generation of protein therapeutics. *Drug Discov. Today* (2008). 10.1016/j.drudis.2008.04.013 18621567

[7] Mouratou B (2007). Remodeling a DNA-binding protein as a specific *in vivo* inhibitor of bacterial secretin PulD. Proceedings of the National Academy of Sciences of the United States of America.

[8] Diem, M. D. *et al*. Selection of high-affinity Centyrin FN3 domains from a simple library diversified at a combination of strand and loop positions. *peds.oxfordjournals.org*10.1093/protein/gzu016 24786107

[9] Carlin, K. & Rao, B. M. *CARLIN, KEVIN BRIAN. Engineering Multivalent Protein Affinity Ligands using the Sso7d Scaffold*. 1–169 (2016).

[10] Bruce VJ, Ta AN, McNaughton BR (2016). Minimalist Antibodies and Mimetics: An Update and Recent Applications. Chembiochem.

[11] Banta S, Dooley K, Shur O (2013). Replacing antibodies: engineering new binding proteins. Annu Rev Biomed Eng.

[12] Dunlevy FK, Martin SL, de Courcey F, Elborn JS, Ennis M (2012). Anti-inflammatory effects of DX-890, a human neutrophil elastase inhibitor. J. Cyst. Fibros..

[13] Binz HK, Amstutz P, Plückthun A (2005). Engineering novel binding proteins from nonimmunoglobulin domains. Nat Biotech.

[14] Vazquez-Lombardi R (2015). Challenges and opportunities for non-antibody scaffold drugs. Drug Discov. Today.

[15] Johannes T. -H. Yeh, Richard Binari, Tenzin Gocha, Ramanuj Dasgupta, Norbert Perrimon, (2013) PAPTi: A Peptide Aptamer Interference Toolkit for Perturbation of Protein-Protein Interaction Networks. Scientific Reports 3 (1). 10.1038/srep01156 PMC3557448 23362456

[16] Gera N, Hussain M, Wright RC, Rao BM (2011). Highly stable binding proteins derived from the hyperthermophilic Sso7d scaffold. J. Mol. Biol..

[17] Traxlmayr, M. W. *et al*. Strong Enrichment of Aromatic Residues in Binding Sites from a Charge-Neutralized Hyperthermostable Sso7d Scaffold Library. *J Biol Chem*10.1074/jbc.M116.741314 (2016). 10.1074/jbc.M116.741314 PMC5077188 27582495

[18] Zhao N, Schmitt MA, Fisk JD (2016). Phage display selection of tight specific binding variants from a hyperthermostable Sso7d scaffold protein library. FEBS J.

[19] Allenspach EJ, Maillard I, Aster JC, Pear WS (2014). Notch Signaling in Cancer. Cancer Biology & Therapy.

[20] Kopan R, Ilagan MXG (2009). The canonical Notch signaling pathway: unfolding the activation mechanism. Cell.

[21] Bray SJ (2016). Notch signalling in context. Nat Rev Mol Cell Biol.

[22] Kitagawa M (2016). Notch signalling in the nucleus: roles of Mastermind-like (MAML) transcriptional coactivators. J. Biochem..

[23] Hambleton S (2004). Structural and Functional Properties of the Human Notch-1 Ligand Binding Region. Structure.

[24] Cordle J (2008). A conserved face of the Jagged/Serrate DSL domain is involved in Notch trans-activation and cis-inhibition. Nat Struct Mol Biol.

[25] Sharma A, Paranjape AN, Rangarajan A, Dighe RR (2012). A Monoclonal Antibody against Human Notch1 Ligand-Binding Domain Depletes Subpopulation of Putative Breast Cancer Stem-like Cells. Molecular Cancer Therapeutics.

[26] Takebe N, Harris PJ, Warren RQ, Ivy SP (2011). Targeting cancer stem cells by inhibiting Wnt, Notch, and Hedgehog pathways. Nat Rev Clin Oncol.

[27] Sharma A, Rangarajan A, Dighe RR (2013). Antibodies against the extracellular domain of human Notch1 receptor reveal the critical role of epidermal-growth-factor-like repeats 25–26 in ligand binding and receptor activation. Experimental Cell Research.

[28] Mittal S, Subramanyam D, Dey D, Kumar RV, Rangarajan A (2009). Cooperation of Notch and Ras/MAPK signaling pathways in human breast carcinogenesis. Mol Cancer.

[29] Luca, V. C., Jude, K. M., Pierce, N. W. & Nachury, M. V. Structural basis for Notch1 engagement of Delta-like 4*. …* (2015). 10.1126/science.1261093 PMC4445638 25700513

[30] Maute RL (2015). Engineering high-affinity PD-1 variants for optimized immunotherapy and immuno-PET imaging. Proceedings of the National Academy of Sciences of the United States of America.

[31] Andrawes MB (2013). Intrinsic Selectivity of Notch 1 for Delta-like 4 Over Delta-like 1. J Biol Chem.

[32] Yamamoto S (2012). A mutation in EGF repeat-8 of Notch discriminates between Serrate/Jagged and Delta family ligands. Science.

[33] Lindsell CE, Shawber CJ, Boulter J, Weinmaster G (1995). Jagged: a mammalian ligand that activates Notch1. Cell.

[34] Shimizu K (1999). Mouse jagged1 physically interacts with notch2 and other notch receptors. Assessment by quantitative methods. J Biol Chem.

[35] Yen WC (2015). Targeting Notch Signaling with a Notch2/Notch3 Antagonist (Tarextumab) Inhibits Tumor Growth and Decreases Tumor-Initiating Cell Frequency. Clinical Cancer Research.

[36] Proia T (2015). 23814, an Inhibitory Antibody of Ligand-Mediated Notch1 Activation, Modulates Angiogenesis and Inhibits Tumor Growth without Gastrointestinal Toxicity. Molecular Cancer Therapeutics.

[37] Lindsey, D. OncoMed. A first-in-human phase I study of the novel cancer stem cell (CSC) targeting antibody OMP-52M51 (anti-Notch1) administered intravenously to patients with certain advanced solid tumors Available at:http://www.oncomed.com/presentations/OMP-52M51%20Ph1a_AACR-NCI-EORTC2013.pdf. (Accessed: 23rd January 2015).

[38] Malmberg J, Tolmachev V, Orlova A (2011). Imaging agents for *in vivo* molecular profiling of disseminated prostate cancer–targeting EGFR receptors in prostate cancer: comparison of cellular processing of [111In]-labeled affibody molecule Z(EGFR:2377) and cetuximab. Int J Oncol.

[39] Natarajan A, Hackel BJ, Gambhir SS (2013). A Novel Engineered Anti-CD20 Tracer Enables Early Time PET Imaging in a Humanized Transgenic Mouse Model of B-cell Non-Hodgkins Lymphoma. Clinical Cancer Research.

[40] Sakamoto K (2012). Reduction of NOTCH1 expression pertains to maturation abnormalities of keratinocytes in squamous neoplasms. Lab Invest.

[41] Wang Y (2004). A novel strategy to engineer DNA polymerases for enhanced processivity and improved performance *in vitro*. Nucleic Acids Res..

[42] Gera N, Hussain M, Rao BM (2013). Protein selection using yeast surface display. Methods.

[43] Baumann H, Knapp S, Lundbäck T, Ladenstein R, Härd T (1994). Solution structure and DNA-binding properties of a thermostable protein from the archaeon Sulfolobus solfataricus. Nat. Struct. Biol..

