# Supplementary material for: Identification and characterization of a novel Sso7d scaffold-based binder against Notch1
Source: Sci Rep. 2017 Sep 20;7:12021. doi: 10.1038/s41598-017-12246-1 (PMC5607287; doi:10.1038/s41598-017-12246-1)
Supplement: Supplementary file 1 — Supplementary files [file 41598_2017_12246_MOESM1_ESM.pdf]

Identification and characterization of a novel Sso7d scaffold-based binder against Notch1

Tenzin Gocha[1], Balaji M. Rao[2]*, Ramanuj DasGupta [1,3]*

[1] New York University Langone Medical center, Perlmutter Cancer Center, Deparment of Biochemistry and Molecular Pharmacology, 522 1st Ave., Smilow Research Building, Rm 1206 New York, NY, 10016, USA

[2]Department of Chemical and Biomolecular Engineering, North Carolina State University, Raleigh, North Carolina, 27695, United States

[3]Genome Institute of Singapore, Cancer Therapeutics and Stratified Oncology, 60 Biopolis Street, #02-01, Genome, 138672, Singapore.

*Correspondence and requests for materials should be addressed to RD (dasguptar@ gis.a-star.edu.sg) or BR (bmrao@ncsu.edu)

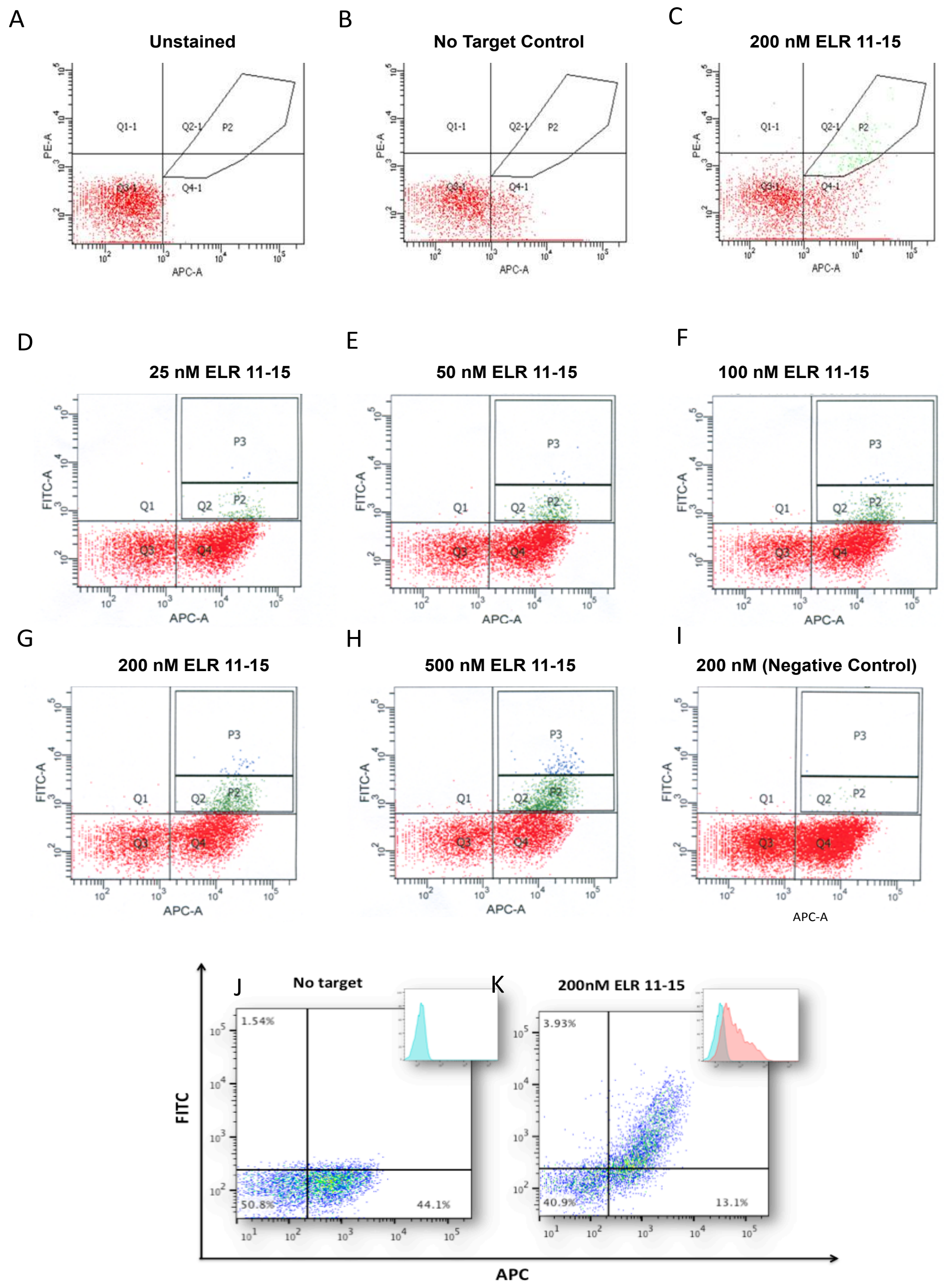

Supplementary Fig. S1. Screening of Notch1 ELR 11-15 sso7d binders. Yeast cells were labeled with biotinylated ELR 11-15 and Chicken anti Myc antibody, followed by labeling with Streptavidin-PE or Neutravidin FITC antibody and Rabbit anti-Chicken APC secondary antibodies. (A) Unstained Yeast (B) No GST ELR 11-15 control (C) FACS screen round1- Post magnetic library labeled at 200nM. Cells in P2 were collected (D-I) FACS Screen Round 2: Cells from round 1 labeled at 25nM, 50nM, 100nM, 200nM, 500nM and a negative control protein 200nM NRR. Cells from 25nM, P3 were collected. (J-K) Sorted cells from Round 2 labeled with no ELR 11-15 and 200nM of ELR 11-15

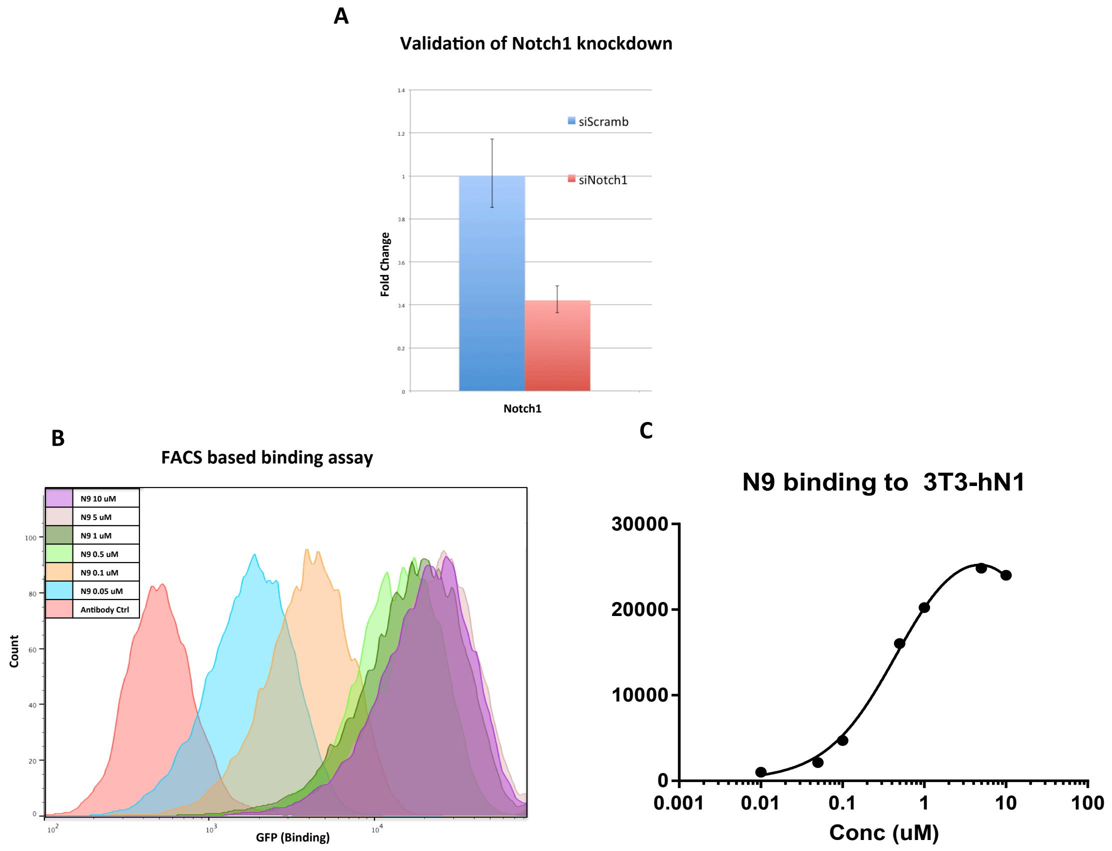

Supplementary Fig. S2. (A) Validation of Notch1 Knockdown in MCF7 cells by qRT-PCR **(B)** Dose dependent binding of N9 on 3T3-hNotch1 cells lines **(C)** Estimation of Kd from mean fluorescence intensity of (B). N9 binds to 3T3-hNtoch1 cells with Kd of ~0.3uM

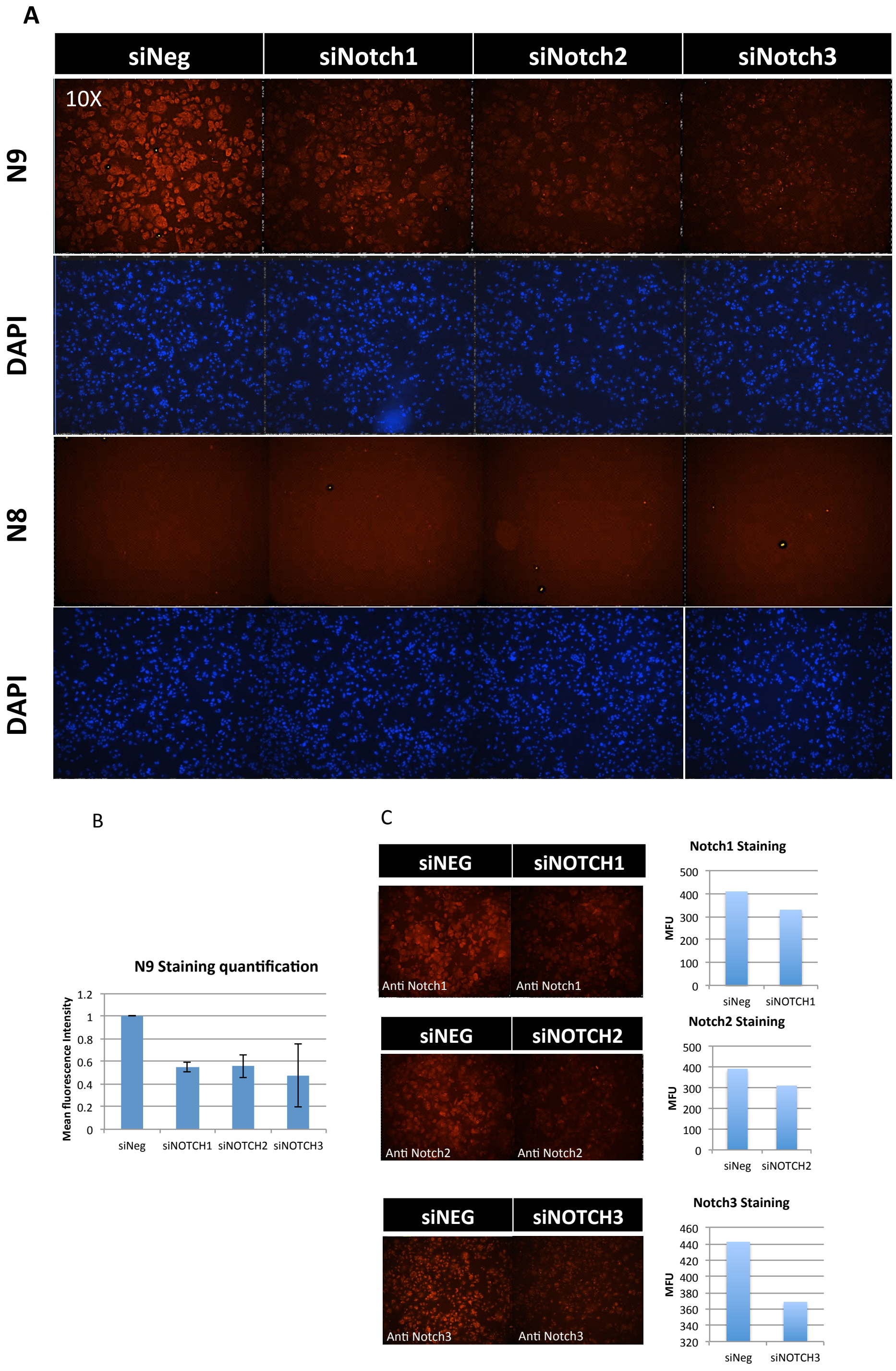

Supplementary Fig. S3. Paralogue specificity of N9. **(A)** Knockdown of a Notch receptors (1-3) decrease staining of N9. Staining with N8 is shown as scaffold control. **(B)** Quantification of N9 staining upon Notch knockdowns. Values from two independent experiments are plotted. **(C)** Validation of knockdown of individual Notch receptors by staining with respective commercial antibodies and their quantifications. Altogether, these show that N9 exhibits cross reactivity with other Notch receptors as well

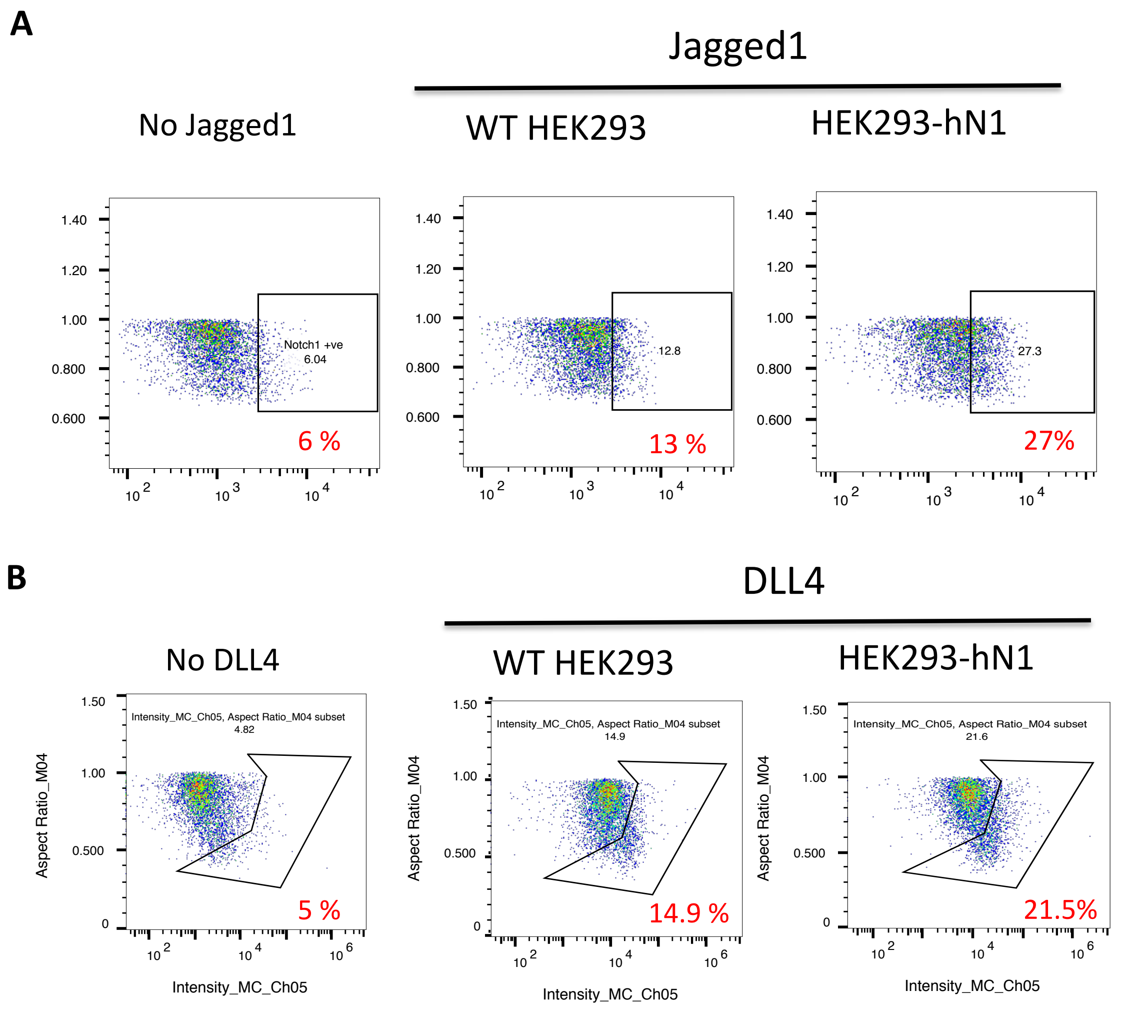

Supplementary Fig. S4. Flow cytometry analysis of JAG1 **(A)** and DLL4 **(B)** binding on hNotch1 transfected HEK293 cells. Note increase in binding of JAG1/DLL4 to HEK293-hN1 cells in comparison to Wild Type HEK293 cell. Wild type HEK293 cells or Notch1 transfected HEK293 cells were incubated with 2nM human recombinant JAG1-Fc or DLL4-Fc (R&D Systems) and incubated for 1 hour. Cells were stained with APC conjugated Mouse Anti-Fc antibody (H2, ab99768) for half hour on ice and analyzed using Amnis ImageStream Imaging flow cytometer.

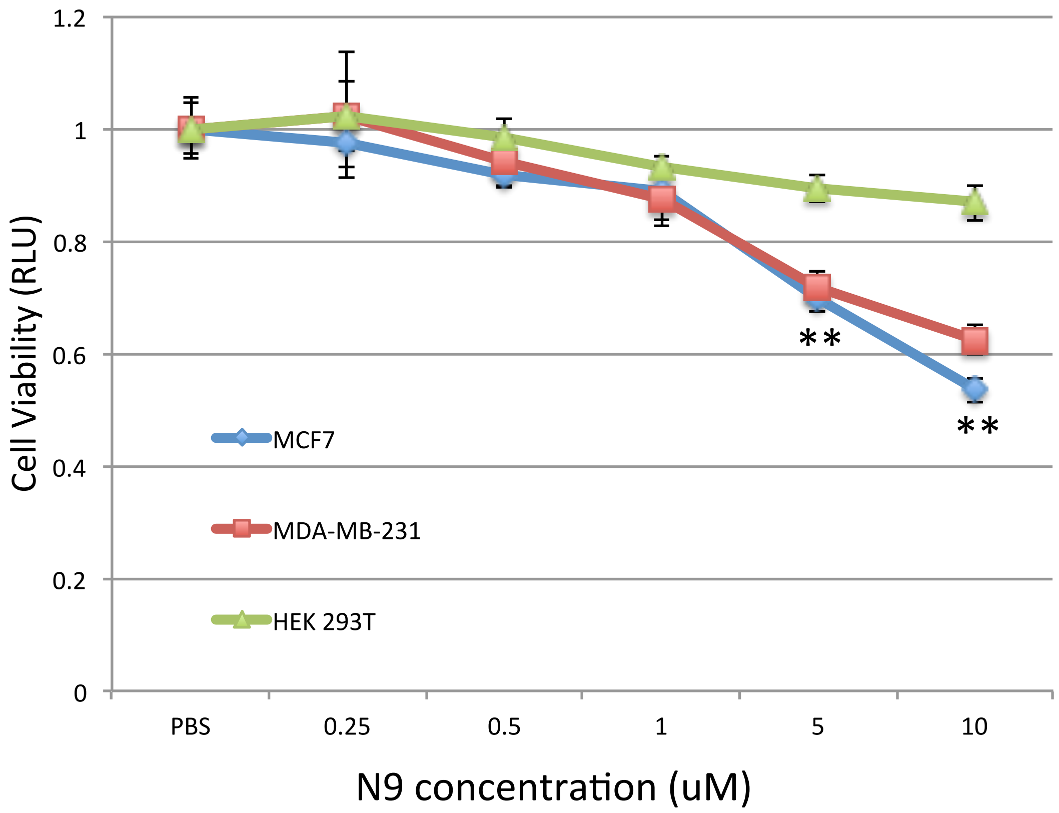

Supplementary Fig. S5. Effect of N9 on cell proliferation. Cultured cells were treated with indicated concentration of N9 and cell viability was measured after 72hrs. N9 doesn't affect proliferation of non-cancerous HEK293 cells, where as proliferations of MCF7 and MDA-MB-231 are reduced. **=p<0.01

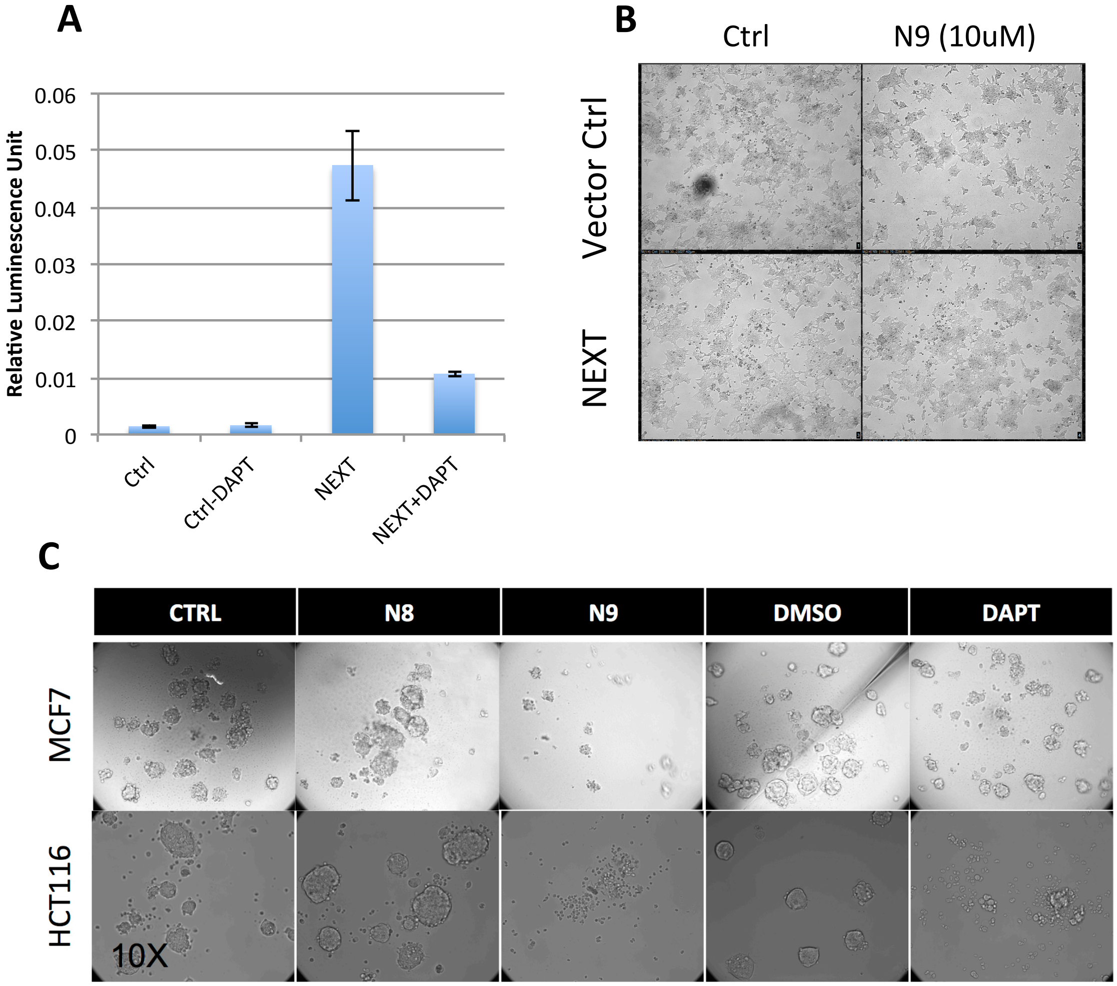

Supplementary Fig. S6. A) Validation of Notch activation with NEXT (Extracellularly truncated Notch). B) Representative image of HCT116 cells after treatment with N9 and rescued with NEXT (Fig 4D). C) Representative images (10X) of MCF7 and HCT116 spheres from Fig.4E and 4F.

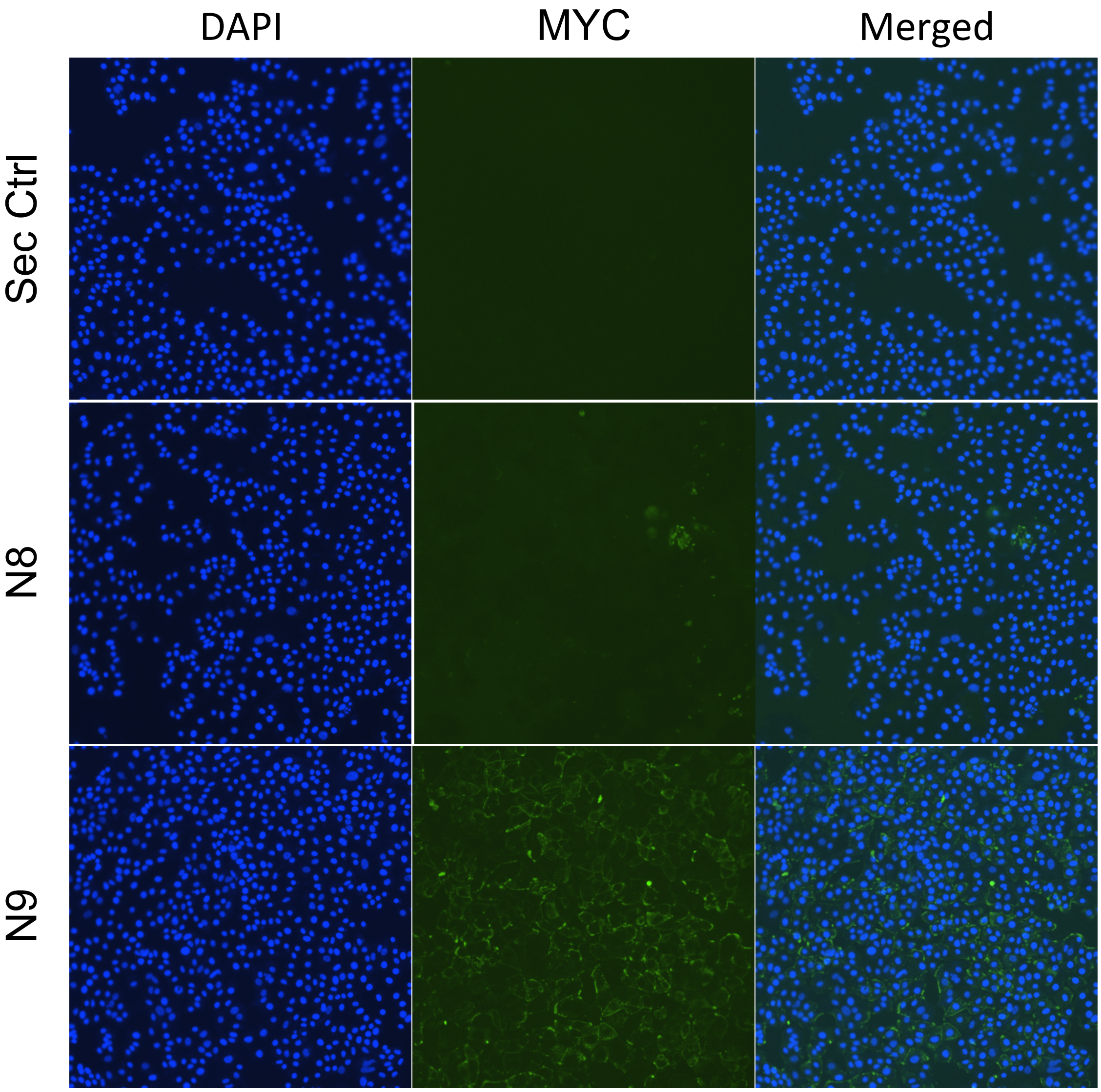

Supplementary Fig. S7. Staining of MCF7 with N8 and N9. MCF7 cells were labeled with Myc tagged N8 and N9 (5uM) and stained with Anti Myc-488 antibody. As seen before, N9, but not N8 binds and stains MCF7 cells

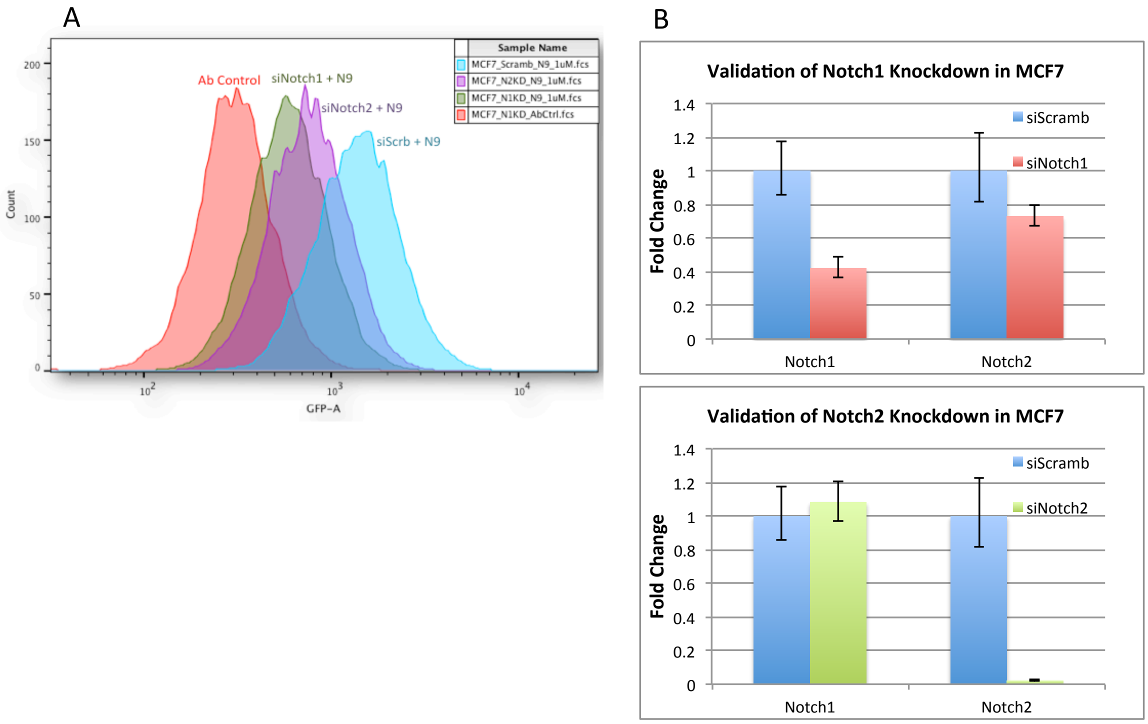

Supplementary Fig. S8. Paralogue specificity of N9. (A) Knockdown of Notch1 as well as Notch2 in MCF7 cells decrease binding of N9. **(B)** qRT PCR validation of Notch1 and Notch2 knockdown

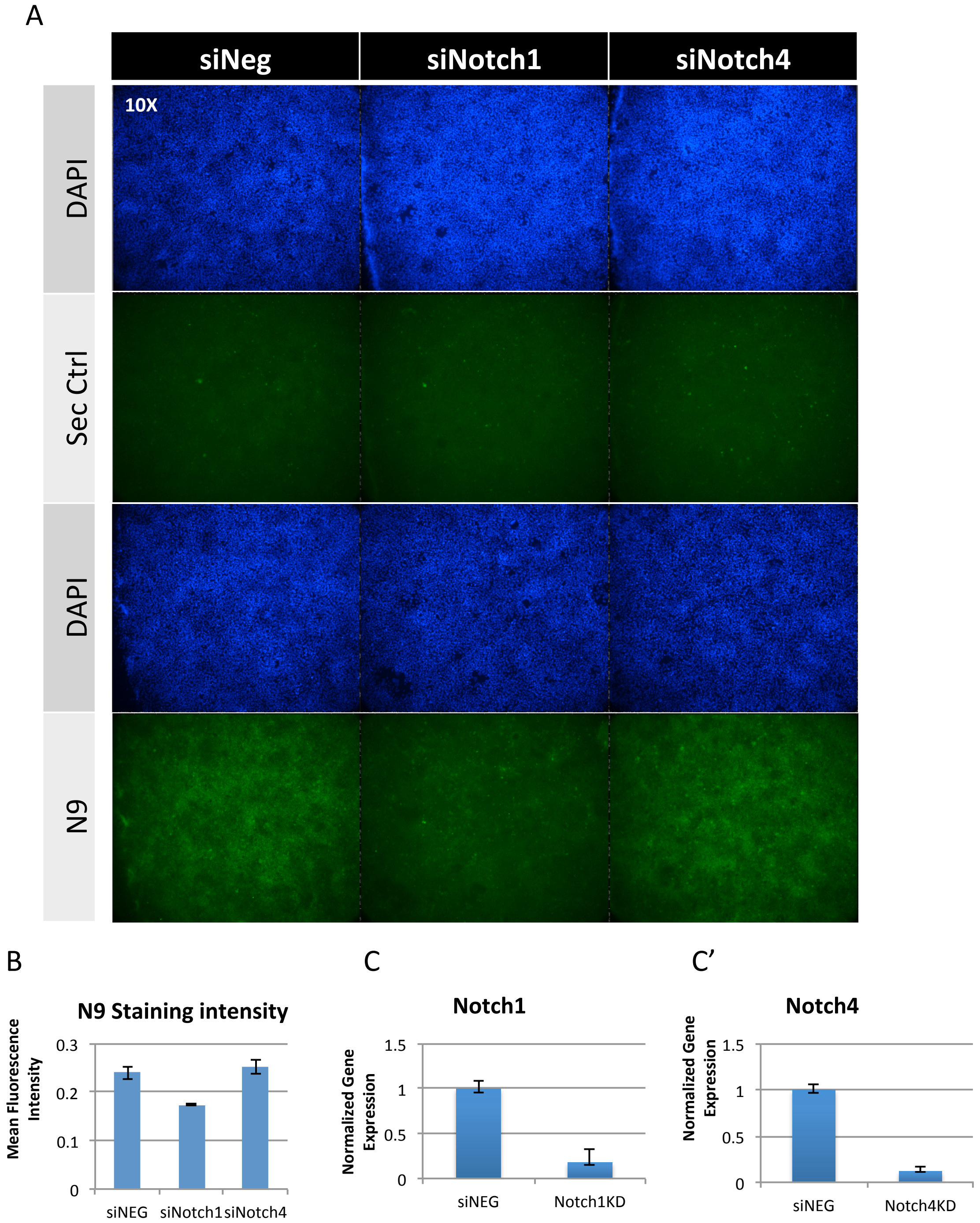

Supplementary Fig. S9. Knockdown of Notch4 has no effect on N9 staining. A) Knockdown of Notch1 but not Notch4 decreases N9 staining. HCT116 cells were transfected with siRNAs against Notch1 and Notch4 and stained with Myc tagged N9 and stained with Anti Myc-488 antibody **B)** Quantification of staining intensity of N9. **C-C')** Validation of knockdown of Notch1 and Notch4 using qPCR

Hes1
Hes1_For: TCAACACGACACCGGATAAAC
Hes1_Rev: GCCGCGAGCTATCTTTCTTCA

GAPDH
GAPDH_For: TGTGGGCATCAATGGATTTGG
GAPDH_Rev: ACACCATGTATTCCGGGTCAAT

Hey1
Hey1_For: GTTCGGCTCTAGGTTCCATGT
Hey1_Rev: CGTCGGCGCTTCTCAATTATTC

Hey2
Hey2_For: AAGGCGTCGGGATCGGATAA
Hey2_Rev: AGAGCGTGTGCGTCAAAGTAG

HeyL
HeyL_For: GGAAGAAACGCAGAGGGATCA
HeyL_Rev: CAAGCGTCGCAATTCAGAAAG

Notch1
Forward: 5 -CTGAAGAACGGGGCTAACAA-3
Reverse: 5 -CAGGTTGTACTCGTCCAGCA-3

Notch2
Forward: 5 -ACCAGTGTGATGAGCTGTGC-3
Reverse: 5 -AGGGTACCTTCTGCCAGGTT-3

Supplementary Table: Primers used for qRT PCR
